# Early Clinical Outcomes of Dual Subsartorial Block Following Total Knee Arthroplasty in an ERAS Protocol: A Descriptive Study

**DOI:** 10.5812/aapm-174150

**Published:** 2026-06-30

**Authors:** Yustin Marinta, Denal Bato Tampak, Ludwig Andre Pontoh, Sholahuddin Rhatomy

**Affiliations:** 1 Department of Orthopedic and Traumatology, Prof. Dr. R. Soeharso Orthopaedic Hospital Jl. Jenderal Ahmad Yani No.157, Pabelan, Kartasura, Sukoharjo, Central Java 57169 57162 Solo Indonesia; 2 Department of Orthopedic and Traumatology,, Bunda Pengharapan Merauke Hospital Tujuh Wali-Wali, Kamundu RT.20/RW.04, Wasur/Kumundu, Kec. Merauke, Papua Selatan. 99616 Merauke Indonesia; 3 Department of Orthopedic and Traumatology, Fatmawati General Hospital, Faculty of Medicine Universitas Indonesia RS. Fatmawati Raya No.4, RT.4/RW.9, Cilandak Barat 12430 Jakarta Indonesia; 4 Department of Orthopaedic and Traumatology, Dr. Soeradji Tirtonegoro General Hospital, Klaten Jl. KRT. dr. Soeradji Tirtonegoro No. 1, Dusun 1, Tegalyoso 57424 Klaten Indonesia

**Keywords:** Total Knee Arthroplasty, Dual Subsartorial Block, Enhanced Recovery After Surgery, Postoperative Pain, Functional Recovery

## Abstract

**Background:**

*Effective postoperative* pain management after total knee arthroplasty (TKA) is essential to facilitate early mobilization and recovery within enhanced recovery after surgery (ERAS) protocols. Dual subsartorial block (DSB) is a regional analgesic technique designed to provide procedure-specific analgesia while potentially preserving motor function.

**Objectives:**

This study aimed to describe postoperative pain, assessed using the visual analog scale (VAS) at 24 and 48 hours, as the primary outcome, and early functional outcomes as secondary outcomes, including performance on the Timed Up and Go test, knee flexion range of motion (ROM), and length of hospital stay, in patients undergoing TKA who received DSB within an ERAS protocol.

**Methods:**

This retrospective descriptive study included 20 patients who underwent primary TKA between August 2021 and January 2022 at Dr. Soeradji Tirtonegoro Hospital, Klaten, Indonesia. Routine clinical records were reviewed for static and dynamic VAS scores at 24 and 48 hours; TUG results and knee flexion ROM on postoperative days 1 and 2; and LOS. Some outcome records were incomplete, and outcome-specific denominators could not be reliably reconstructed from the archived records. Available values are summarized descriptively; missing values were not imputed.

**Results:**

Among the values available in the records, the mean static VAS score was 2.26 ± 1.098 at 24 hours and 1.53 ± 0.964 at 48 hours; the mean dynamic VAS score was 3.47 ± 1.504 and 2.21 ± 1.084, respectively. Mean Timed Up and Go time was 53.501 ± 12.269 seconds on postoperative day 1 and 48.968 ± 13.609 seconds on day 2. Mean knee flexion ROM was 75.25° ± 18.244 on day 1 and 96.25° ± 13.365 on day 2. Mean length of hospital stay was 3.11 ± 1.449 days. Because outcome-specific denominators were not recoverable and participant-level paired data were unavailable, no inferential comparisons between time points are reported.

**Conclusions:**

Patients receiving DSB within an ERAS protocol had low postoperative pain scores and showed improvements in early functional measures during the assessed postoperative period. Because this was a descriptive study without a control group, the observed outcomes cannot be attributed specifically to DSB. *Prospective comparative* studies with larger sample sizes are needed to evaluate the effectiveness of DSB relative to other postoperative analgesic techniques.

## 1. Background

Total knee arthroplasty (TKA) is an established surgical treatment for end-stage knee osteoarthritis, and its use continues to increase alongside the growing burden of degenerative knee disease (1). As the number of TKA procedures increases, optimization of perioperative care, efficient resource utilization, and reduction of health care costs have become increasingly important (2). Strategies to accelerate postoperative recovery and reduce unnecessary length of hospital stay (LOS) have therefore become important components of contemporary TKA management (1, 2).

Over the past two decades, perioperative management of patients undergoing hip and knee arthroplasty has progressively shifted toward fast-track and enhanced recovery after surgery (ERAS) protocols. ERAS is a multimodal, evidence-based approach designed to reduce surgical stress, optimize perioperative care, facilitate early mobilization, and accelerate postoperative recovery (2, 3).

*Effective postoperative* pain management is an important component of ERAS after TKA. Multimodal analgesic strategies combine nonopioid pharmacological therapies and regional analgesic techniques to provide adequate pain control while minimizing opioid requirements and associated adverse effects (2-4). Paracetamol and nonsteroidal anti-inflammatory drugs are commonly included in multimodal analgesic regimens, while peripheral nerve blocks may provide additional procedure-specific analgesia (2-4).

Femoral nerve block has traditionally been used to provide postoperative analgesia after TKA. Although it can provide satisfactory pain control, associated quadriceps weakness may interfere with early mobilization and potentially increase the risk of falls (5-7). Adductor canal block has subsequently emerged as a more motor-sparing alternative, although its analgesic coverage may vary (8).

Dual subsartorial block (DSB) has recently been introduced as a procedure-specific regional analgesic technique for TKA. The technique combines injections at two subsartorial locations to provide analgesic coverage of structures contributing to postoperative knee pain. Previous studies have reported motor-sparing and opioid-sparing characteristics of DSB (9, 10). These characteristics may be relevant to ERAS pathways, in which adequate analgesia and early mobilization are important components of postoperative recovery. However, motor function was not directly evaluated in the present study.

## 2. Objectives

Evidence regarding postoperative outcomes after DSB remains limited, particularly in Indonesia. Therefore, this study aimed to describe postoperative pain and early functional outcomes in patients undergoing TKA who received DSB within an ERAS protocol.

## 3. Methods

### 3.1. Study Design

This was a retrospective descriptive observational study of routine clinical records for patients who underwent primary TKA between August 2021 and January 2022 at Dr. Soeradji Tirtonegoro Hospital, Klaten, Indonesia. Records were reviewed for postoperative pain and early functional outcomes. The study was descriptive and had no comparison group.

Patients were eligible if the available records indicated all of the following: age ≥ 50 years; grade IV knee osteoarthritis treated with primary TKA using a medial parapatellar approach under spinal anesthesia; and receipt of postoperative DSB involving a distal femoral triangle block and an adductor canal block. Patients were excluded if the records indicated an additional regional anesthetic procedure, such as a femoral nerve block, or a known allergy to ropivacaine. Twenty patients were included based on the available records. The participant flow summary, based on the Strengthening the Reporting of Observational Studies in Epidemiology (STROBE) guidelines, is as follows: 20 patients met the eligibility criteria in the available records, and all 20 were included and analyzed; none were lost to follow-up within the reporting window because only recorded data were used. The original screening log and complete historical screening records were unavailable; therefore, the number of patients screened, excluded for each reason, or otherwise not included could not be reconstructed, and no screening flow diagram could be produced. The number of missing observations for each outcome and time point could not be determined from the archived records, and no missing values were imputed; therefore, summaries are based on the values that were available. The eligibility criteria were selected to describe a relatively consistent group receiving the DSB protocol while excluding concomitant regional blockade that could complicate interpretation.

### 3.2. Study Participants

The study included patients who underwent primary TKA between August 2021 and January 2022. The inclusion criteria were as follows: 1) age ≥ 50 years; 2) grade IV knee osteoarthritis treated with primary TKA using a medial parapatellar approach under spinal anesthesia; and 3) receipt of postoperative DSB consisting of a distal femoral triangle block and an adductor canal block.

Patients were excluded if they met either of the following criteria: 1) undergoing additional regional anesthetic procedures, such as a femoral nerve block; or 2) having a known allergy to ropivacaine.

Twenty patients were included based on the available clinical records. The original screening log and complete historical screening records were unavailable; therefore, a complete screening flow and counts for individual exclusion reasons could not be reported.

An initial sample size estimate was reportedly calculated using a single-proportion formula with a 95% confidence level and a margin of error of 0.2. A proportion of P = 0.44 was used in that calculation, but its source could not be verified from the available records; therefore, the calculation is reported as unverified and cannot be independently reproduced. It is not used to justify precision or inferential conclusions, and the final sample was determined by the number of available eligible records rather than by the calculation. The corresponding limitation is stated in the Discussion. The analysis is treated as a descriptive review of the 20 eligible records available for this study; no claim is made that the sample size was outcome specific or statistically powered.

### 3.3. Patient Preparation and Dual Subsartorial Block Technique

The available clinical records documented postoperative ultrasound-guided DSB involving the distal femoral triangle and adductor canal. The following anatomical description outlines the intended DSB approach; the retrospective records did not allow independent verification of every technical detail for each patient.

A total of 20 mL of 0.2% ropivacaine combined with 8 mg of dexamethasone was used and divided equally between two injection sites. The first 10-mL injection was administered at the distal femoral triangle. With the patient supine and the hip slightly abducted and externally rotated, a linear ultrasound probe was placed transversely over the anteromedial aspect of the thigh. The apex of the femoral triangle was identified as the intersection of the medial borders of the sartorius and adductor longus muscles, an appearance described in the DSB literature as the "figure-of-three" or "kissing sign" (9, 10). Local anesthetic was deposited in the subsartorial fascial plane deep to the sartorius muscle and superficial to the vastus medialis muscle, targeting the saphenous nerve and the nerve to the vastus medialis. The second 10-mL injection was administered at the adductor canal, identified as the fascial compartment bounded superficially by the sartorius muscle, anterolaterally by the vastus medialis muscle, and posteriorly by the adductor longus and adductor magnus muscles, with the femoral artery and its accompanying femoral vein serving as the key intracanal sonoanatomical landmarks (Figure 1A, Figure 2B). Local anesthetic was distributed around the saphenous nerve and subsartorial plexus adjacent to the femoral artery within the canal. This second injection was intended to permit distal spread of local anesthetic toward the popliteal plexus through the adductor hiatus, thereby providing posterior knee analgesia (9, 10).

**Figure 1.**
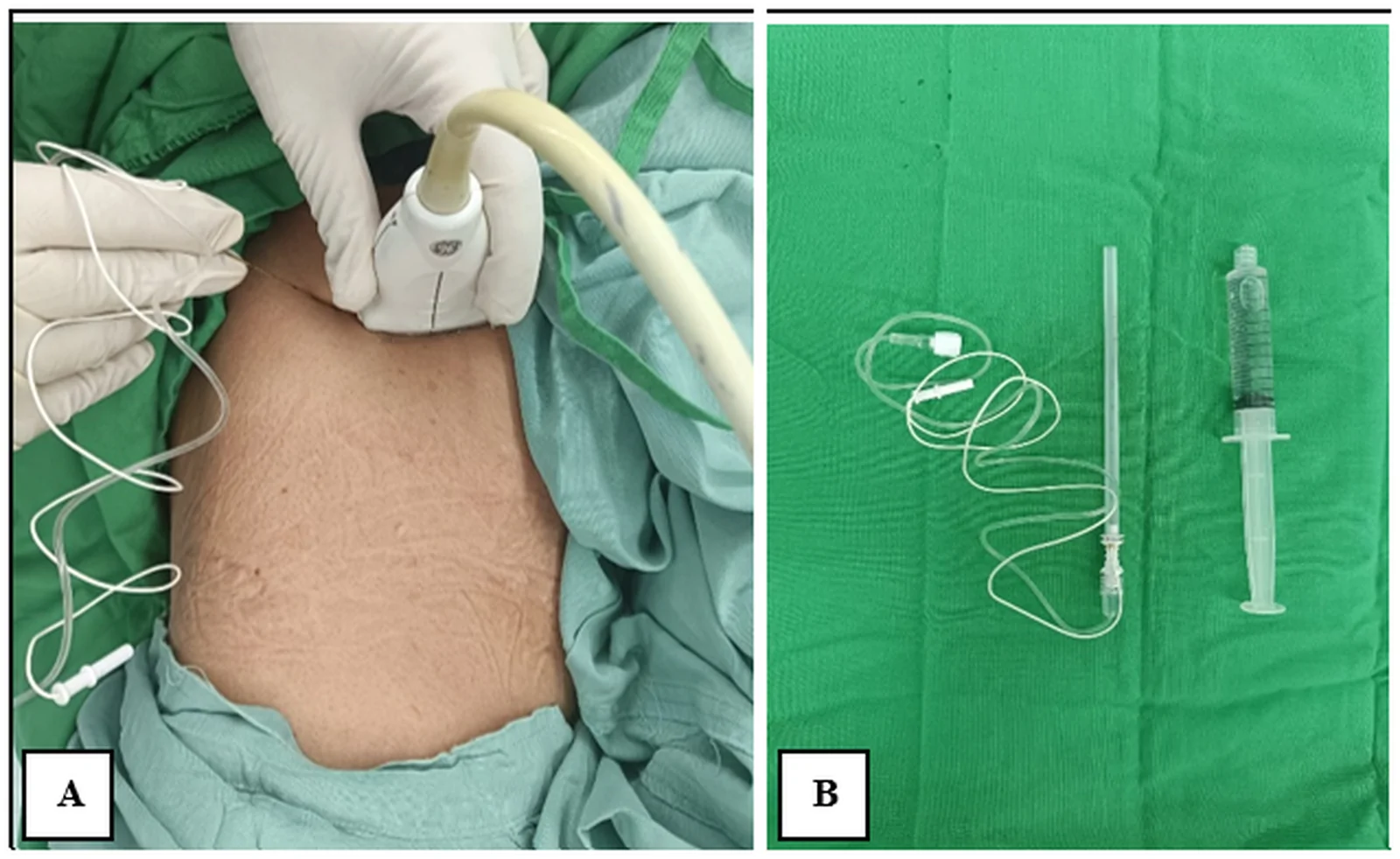
Probe Position and Equipment. A, A high-frequency linear-array ultrasound probe placed horizontally over the anteromedial aspect of the thigh reveals the prominent muscles (sartorius, vastus medialis, adductor longus) of the thigh. The femoral vessels lie beneath the sartorius muscle and can be identified using color Doppler for orientation. B, Local anesthetic solution: 20 mL (0.2% ropivacaine + 8 mg dexamethasone).

**Figure 2.**
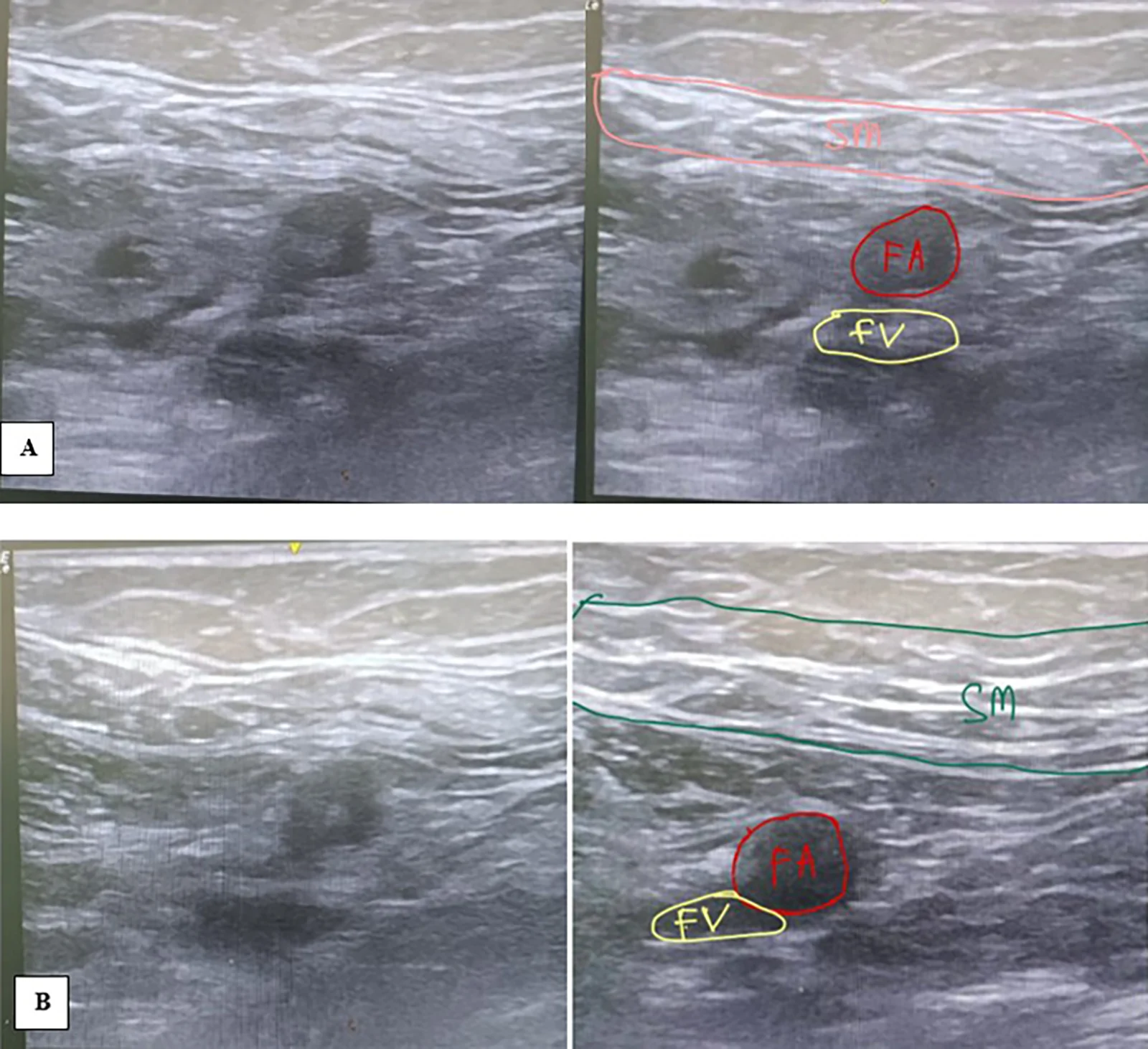
Ultrasound-Guided Dual Subsartorial Block. A, Distal femoral triangle block demonstrating the sartorius muscle overlying the femoral artery and femoral vein; local anesthetic is deposited in the subsartorial fascial plane adjacent to the femoral artery. B, Adductor canal block demonstrating the SM over the FA and FV within the adductor canal. Local anesthetic is injected beneath the sartorius muscle to anesthetize the saphenous nerve.

The following procedural elements were documented in the available records and are therefore reported as performed: ultrasound-guided needle advancement with real-time visualization; a single-injection technique at each of the two sites; a total volume of 20 mL of 0.2% ropivacaine with 8 mg of dexamethasone, divided into two 10-mL injections; the two anatomical target sites (distal femoral triangle and adductor canal); and performance of the block by an experienced orthopedic surgeon immediately after surgery. The archived records did not consistently document several other technical details, including needle gauge and type, aspiration before injection, incremental injection technique, confirmation of local-anesthetic spread, and the exact timing of the block relative to tourniquet release. These undocumented steps are therefore not presented as verified procedures performed in every case, and no procedural details beyond those listed above should be assumed.

Routine postoperative clinical records were reviewed for pain scores at 24 and 48 hours, functional assessments when recorded, analgesic treatment, and documented adverse events. The usual postoperative regimen included scheduled paracetamol and ketorolac, with tramadol available as rescue analgesia when clinically indicated, alongside rehabilitation under the hospital's ERAS pathway. Documentation of rescue-tramadol administration and safety events was incomplete. Specifically, no adverse events (block failure, falls, clinically apparent motor weakness, neurological complications, or signs of local anesthetic systemic toxicity) were identified in the available records, and the available records do not permit quantification of rescue-tramadol administration. Whether these outcomes were systematically recorded could not be determined; therefore, a zero event rate cannot be reported, and the absence of a recorded event should not be interpreted as proof that no event occurred.

### 3.4. Clinical Assessment

#### 3.4.1. Primary Outcome: Postoperative Pain

Pain intensity was assessed as part of routine clinical care using the visual analog scale (VAS; 0 - 10) at 24 and 48 hours postoperatively, when recorded. Static VAS scores referred to pain at rest, and dynamic VAS scores referred to pain during movement (11). The archived data did not permit reliable reconstruction of the number of available observations at each time point.

#### 3.4.2. Secondary Outcomes

Functional mobility was assessed as part of routine clinical care using the Timed Up and Go (TUG) test on postoperative days 1 and 2 with a walker, when recorded (12). The documented procedure involved rising from a seated position on instruction, walking 3 m with the walker, turning, returning to the chair, and sitting. The records did not allow independent verification of whether all elements were standardized consistently across assessments.

Length of hospital stay was recorded from the day of surgery until hospital discharge (13).

Knee flexion range of motion (ROM) was measured using a goniometer on postoperative days 1 and 2, when recorded (14). The clinical documentation described a supine position and alignment of the goniometer with the anatomical axis of the knee; however, the retrospective records did not allow confirmation that the measurement procedure was identical for every assessment. The following measurement details could not be verified from the records: the assessors and their training, the number of TUG trials performed per assessment, the exact chair and walking-course setup, the timing of assessments relative to scheduled analgesia, and whether the same goniometer and positioning were used for every ROM measurement. These elements should be considered unverified rather than standardized.

### 3.5. Data Analysis

Data were analyzed using SPSS version 25.0. Descriptive statistics are reported for the available recorded values. Categorical characteristics are summarized as frequencies and percentages based on the documented cohort (n = 20). Continuous variables are presented as mean ± standard deviation (SD) where available. All available recorded outcome values were used, and no missing values were imputed. Because some outcome records were incomplete and outcome-specific denominators could not be reconstructed, the number of observations contributing to each outcome summary cannot be reliably stated; readers should assume that each summary may be based on fewer than 20 observations. Tables state that the denominators are not recoverable. Interpretation of the paired changes is therefore descriptive only, as reported in the Results. Confidence intervals and inferential comparisons were not retained because their calculation could not be verified against outcome-specific denominators and participant-level data.

The original participant-level paired data were not available for reanalysis. Therefore, changes between time points were not tested statistically, and no P values are reported. Results are presented descriptively only. The study design, incomplete outcome records, unavailable screening logs, and absence of a control group preclude causal conclusions about DSB.

### 3.6. Ethical Considerations

All assessments reported in this study (VAS, TUG, knee flexion ROM, LOS, analgesic administration, and adverse-event documentation) were performed as part of routine clinical care, and data were extracted from routine clinical records; no study-specific assessments were performed. Because the decision to analyze and publish these previously collected data was made after the data collection period had ended, ethical review was subsequently obtained from the Research Ethics Committee of Dr. Soeradji Tirtonegoro General Hospital, Klaten, Indonesia (No. DP.04.03/D.XXVI.10.6/11976/2026, dated June 25, 2026, valid until June 25, 2027) before manuscript preparation and submission. The committee determined that the study was ethically acceptable in accordance with the seven World Health Organization (WHO) 2011 ethical standards and the 2016 Council for International Organizations of Medical Sciences Guidelines, including standards relating to confidentiality, privacy, and informed consent. All patient data were anonymized before analysis, and the study was conducted in accordance with the principles of the Declaration of Helsinki.

## 4. Results

A total of 20 patients were included in the study. Fifteen patients (75%) were female, and 5 (25%) were male. The mean age was 64.16 ± 7.819 years. Diabetes mellitus was the most frequently recorded comorbidity, occurring in 2 patients (10%) (Table 1). Patient characteristics, including gender, comorbidities, history of previous surgery, and the affected knee side, are summarized in Table 1.

**Table 1.** Patient Characteristics ^a^

Patient Characteristic	Values
**Number of patients**	20
**Gender**	
Male	5 (25.00)
Female	15 (75.00)
**Mean age, y**	64.16 ± 7.819 (60.39 - 67.93)
**Comorbidities**	
Diabetes mellitus	2 (10.0)
Hypertension	1 (5.0)
Anemia	1 (5.0)
**Previous surgery history**	
Surgery for myoma	1 (5.0)
Total knee arthroplasty	3 (15.0)
Surgery for breast cancer	1 (5.0)
**Affected knees**	
Right knee	11 (55.00)
Left knee	2 (10.00)
Both knees	7 (35.00)

^a^ Values are expressed as No. (%) or mean ± SD (95% CI). Abbreviations: CI, confidence interval; SD, standard deviation; TKA, total knee arthroplasty.

Among the available values, the mean static VAS score was 2.26 ± 1.098 at 24 hours and 1.53 ± 0.964 at 48 hours. The mean dynamic VAS score was 3.47 ± 1.504 at 24 hours and 2.21 ± 1.084 at 48 hours. These descriptive summaries are retained from the available analysis, but outcome-specific denominators could not be verified and should be interpreted cautiously.

The mean TUG time was 53.501 ± 12.269 seconds on postoperative day 1 and 48.968 ± 13.609 seconds on postoperative day 2. Mean knee flexion ROM was 75.25° ± 18.244 on day 1 and 96.25° ± 13.365 on day 2. The mean LOS was 3.11 ± 1.449 days. Because the denominators and underlying patient-level data could not be confirmed, confidence intervals and paired comparisons are not reported.

Patient characteristics and clinical outcomes are summarized in Tables 1 and 2, respectively. Some outcome observations were missing from the archived clinical records, and the number of available observations for each outcome and time point could not be reconstructed. No missing values were imputed. Therefore, the results provide descriptive summaries of the available records rather than complete estimates for all 20 patients.

**Table 2.** Clinical Outcomes ^a^

Variables	Values
**Operated knee (TKA procedure)**	
Right knee	15 (75.00)
Left knee	5 (25.00)
**Static VAS (0 - 10), h**	
24	2.26 ± 1.098
48	1.53 ± 0.964
**Dynamic VAS (0 - 10), h**	
24	3.47 ± 1.504
48	2.21 ± 1.084
Length of stay, d	3.11 ± 1.449
**TUG test with walker, s**	
Day 1	53.501 ± 12.269
Day 2	48.968 ± 13.609
**Knee flexion ROM, degree**	
Day 1	75.25 ± 18.244
Day 2	96.25 ± 13.365

^a^ Values are expressed as No. (%) or mean ± SD. Outcome summaries are based on available routine records. Outcome-specific denominators could not be reconstructed; no missing values were imputed. Abbreviations: ROM, range of motion; TKA, total knee arthroplasty; TUG, Timed Up and Go; VAS, visual analog scale.

## 5. Discussion

This descriptive study characterized postoperative pain and early functional outcomes in patients undergoing TKA who received DSB within an ERAS protocol. During the first 48 postoperative hours, patients demonstrated relatively low pain scores, while TUG performance and knee flexion ROM improved between postoperative days 1 and 2. These observations describe the early postoperative course of this cohort but should not be interpreted as evidence of a causal effect of DSB.

DSB is designed to provide procedure-specific analgesic coverage through injections at two subsartorial locations. The first injection targets structures including the saphenous nerve and nerve to the vastus medialis, whereas the second injection, at the adductor canal, is intended to provide additional subsartorial coverage and distal spread toward the popliteal plexus (9, 10). Previous investigations have described DSB as a potentially motor-sparing and opioid-sparing technique following TKA (9, 10).

In the present study, the mean static VAS score was 2.26 ± 1.098 at 24 hours and 1.53 ± 0.964 at 48 hours, and the mean dynamic VAS score was 3.47 ± 1.504 at 24 hours and 2.21 ± 1.084 at 48 hours. Previous DSB studies have similarly reported relatively low postoperative pain scores following TKA (9, 10). Nevertheless, the duration of analgesia attributable to a single DSB cannot be determined from the present data. Consistent with the pharmacology of a single-injection regional technique, the lower pain scores at 48 hours should not be interpreted as evidence of sustained DSB analgesia, because a decline in block effect over time is expected. The observed pain trajectory may also have been influenced by multimodal postoperative analgesia, rehabilitation, natural postoperative recovery, and other perioperative factors.

The rationale for DSB within an ERAS pathway relates not only to analgesic coverage but also to its reported motor-sparing characteristics (9, 10). Preservation of motor function could theoretically facilitate early mobilization following TKA. However, motor strength was not directly measured in the present study. Consequently, improvement in TUG performance should not be interpreted as evidence that DSB preserved quadriceps or lower-extremity motor function.

Mean LOS in the present cohort was 3.11 ± 1.449 days. Agarwala et al. reported a mean hospital stay of approximately 3.98 days in patients undergoing TKA within an enhanced recovery setting (15). Although the LOS observed in the present study was numerically shorter, direct comparison is limited by differences in study population, perioperative protocols, healthcare settings, and study design. Furthermore, because the present study lacked a comparison group, the observed LOS cannot be attributed specifically to DSB.

Functional mobility, assessed using the TUG test with a walker, improved from a mean of 53.501 ± 12.269 seconds on postoperative day 1 to 48.968 ± 13.609 seconds on postoperative day 2. Early mobilization is an important component of ERAS pathways following joint arthroplasty and has been associated with improved postoperative recovery and reduced complications (16, 17). However, the improvement observed in this cohort may reflect several factors, including rehabilitation, postoperative analgesia, increasing patient confidence with ambulation, and the natural postoperative recovery trajectory.

Knee flexion ROM increased from 75.25° ± 18.244 on postoperative day 1 to 96.25° ± 13.365 on postoperative day 2. Oka et al. reported that early postoperative knee flexion ROM may be associated with subsequent recovery of knee flexion following TKA (14). Nevertheless, the observed increase in ROM in the present study cannot be attributed specifically to DSB, because rehabilitation, pain control, postoperative recovery, and other perioperative factors may also have contributed.

Overall, the present findings provide descriptive information regarding early postoperative pain, mobility, knee flexion ROM, and LOS in patients receiving DSB within an ERAS protocol. These findings may provide preliminary clinical data to inform the design of future comparative studies. However, they do not establish the effectiveness or superiority of DSB compared with other regional analgesic techniques.

### 5.1. Study Limitations

This study has several limitations. First, the sample size was relatively small, limiting the precision and generalizability of the findings. Second, this was a single-center study, which may limit external validity and may introduce selection bias. Third, the absence of a control group prevented direct comparison between DSB and other analgesic techniques and did not allow the observed postoperative changes to be distinguished from the natural recovery trajectory following TKA. Fourth, the short follow-up period limited evaluation of longer-term pain and functional outcomes. Fifth, motor strength was not objectively assessed; therefore, the motor-sparing characteristics of DSB could not be evaluated directly. Finally, postoperative analgesic consumption was not evaluated, preventing assessment of the opioid- or analgesic-sparing effect of DSB. In addition, the original screening log was unavailable, some routine outcome records were incomplete, and outcome-specific denominators could not be reconstructed. The source of the sample size proportion used initially (P = 0.44) could not be verified. Participant-level paired data were unavailable for reanalysis, so time-point comparisons and their P values were removed. Rescue-analgesia administration and safety-event documentation were also incomplete; consequently, no reliable rates can be reported and unrecorded events cannot be assumed not to have occurred. These limitations reduce the precision and completeness of the descriptive summaries.

Future prospective comparative studies with larger sample sizes, standardized analgesic protocols, objective motor-strength assessment, analgesic consumption data, and appropriate comparison groups are needed to determine the effectiveness of DSB following TKA.

### 5.2. Conclusions

Patients undergoing TKA who received DSB within an ERAS protocol demonstrated low postoperative pain scores and improvements in TUG performance and knee flexion ROM during the early postoperative period. However, because of the descriptive study design, small sample size, absence of a control group, and potential influence of other perioperative factors, these findings cannot establish that DSB caused the observed outcomes. *Prospective comparative* studies are required to determine the effectiveness of DSB relative to other postoperative analgesic techniques.

## Data Availability

The data presented in this study are uploaded during submission as a supplementary file and are openly available for readers upon request.
